# Good jobs, good pay, better health? The effects of job quality on health among older European workers

**DOI:** 10.1007/s10198-017-0867-9

**Published:** 2017-01-13

**Authors:** Golo Henseke

**Affiliations:** UCL Institute of Education, London, UK

**Keywords:** Job quality, Older workers, Physical health, Mental health, Selection, Panel, Health dynamics, I14, I12, J81

## Abstract

Using data from the Survey of Health, Ageing and Retirement in Europe, this study presents new evidence on the effects of job quality on the occurrence of severe acute conditions, the level of cardiovascular risk factors, musculoskeletal disorders, mental health, functional disabilities and self-assessed health among workers aged 50+. By combining intrinsic job quality with job insecurity and pay the study maps out multiple potential pathways through which work may affect health and well-being. Levering longitudinal data and external information on early retirement ages allows for accounting of unobserved heterogeneity, selection bias and reverse causality. The empirical findings suggest that inequities in health correlate with inequities in job quality, though a substantial fraction of these associations reflect time-constant unobserved heterogeneity. Still, there is evidence for genuine protective effects of better jobs on musculoskeletal disorders, mental health and general health. The effect could contribute to a substantial number of avoidable disorders among older workers, despite relatively modest effect sizes at the level of individuals. Mental health, in particular, responds to changes in job quality. Selection bias such as the healthy worker effect does not alter the results. But the influence of job quality on health may be transitional among older workers. An in-depth analysis of health dynamics reveals no evidence for persistence.

## Introduction

This paper examines the effects of job quality on physical and mental health for a population of workers aged 50–65 between 2004 and 2013 in 15 European countries using data from the Survey of Ageing, Health and Retirement in Europe (SHARE).

Despite improving morbidity and mortality, an almost universal access to health care, health and safety regulations, and an overall decline in accidents at work, around a quarter of Europeans believe their jobs to be pathogenic [1]. The current cost of work-related morbidities is estimated to amount to 2.6–3.8% of annual EU GDP [2]. Moreover, multiple studies have confirmed that poor job quality influences retirement intentions [3], and contributes to early cessation of labour force participation [4, 5], which could partly be driven by work-related ill-health [e.g. 6, 7].

On the one hand, extended working lives, a largely unbroken trend to more intense work [8], and increasingly common precarious employment relations [9] have an impact on job quality and potentially add to work-related health hazards. On the other hand, improvements in work-life balance, greater skills utilisation, and reduced exposure to physical and environmental hazards have made workplaces safer and more flexible to the needs of employees. Within this context, the current study examines how job quality affects health and how these effects shape health over time in a general population of workers aged 50 and older in European countries. In doing so, this paper contributes to the growing economic literature on work and well-being.

Job quality is thought to affect health mainly through exposure to work stress. Situations in which job demands exceed the resources available to the individual—physical capacity, knowledge, psychosocial rewards or decision latitude—to cope with the demands are conjectured to be stressors with potentially adverse health effects [10–12]. The negative consequences from continuous or repeated exposure to even minor work-related stressors can accumulate over time, cause dysregulation in the body and eventually lead to health problems [13]. Environmental hazards at work, psychosocial facets of job quality and material well-being have been found to correlate with a range of stress-related physical and mental health disorders [14, 15].

But while existing models of work stress have greatly helped to conceptualise a link between work and health, psychosocial job facets cover only a fraction of wider job quality. Building on interdisciplinary evidence, Green and Mostafa [16] and Green et al. [8] have proposed a model of objective job quality that combines a more comprehensive list of potentially health and well-being related job facets. Their model covers earnings, intrinsic job quality, job prospects and working time quality, which may affect physical and psychological well-being through stress or material positioning. Job prospects, in particular, may be of growing significance as non-standard employment situations are becoming more widespread in Europe [17, 18]. By focusing on objective job characteristics, Green et al.’s approach avoids the use of hard to compare expressions of job satisfaction that may change with individual aspirations, expectations or personalities. These objective job quality domains are thought to satisfy workers’ needs and endow individuals with the capability to “do and be things they value” [16]. The underlying principles have for example been used to map out the distribution of job quality in European countries [19, 20] or the gender gradient in work-related health [21]. Unlike psychosocial theories of work stress, Green et al.’s concept does not theorise on specific configurations of working conditions that create health risks (e.g. job strain or effort-reward imbalances), but argues that the satisfaction of needs will influence well-being. In the following, I will apply their job quality concept to examine job-facet specific health impacts. A clearer understanding of which job facet relates to which health outcomes will illuminate in greater detail the relation of work to well-being. In doing so, this study analyses the effects of everyday working conditions for the average older workers and not the consequences of specific work-related risk factors in narrowly defined fractions of the workforce.

A growing body of economic research has raised concerns about the causal nature of the association between job facets and health outcomes (see Barnay [1] for a recent review). Firstly, if workers with greater health risks sort into more stressful jobs, the estimated effects of poor job quality on health may be spurious. This is referred to as unobserved heterogeneity and arises from insufficient information on the determinants of job assignment. The concurrent labour market positioning, type of job and health trajectory will depend on events and circumstances throughout the life-course [22–26]. In effect, people from better off socio-economic backgrounds tend to be in better health and employed in higher quality jobs [27, 28]. Secondly, work-related health impairments could lead to early labour force withdrawal and thus to an endogenous sample composition. Especially, workers who are eligible for early retirement pensions may opt for economic inactivity rather than further exposure to adverse working conditions and risk of health deteriorations [29]. Thirdly, health and work may be simultaneously determined in a bidirectional relationship. Work affects health, but health also influences labour supply and job characteristics such as wages [30, 31]. If this is true, a fraction of the cross-sectional association between job quality facets and health may reflect reverse causality.

Health is also highly persistent over the lifecycle [32, 33]. Health shocks may thus develop a multiplicative effect as transitional spells of ill-health evolve into chronic conditions. If work-related health deteriorations persist over time, even relatively modest short-term gains will help to improve individual health and well-being in the long-run. Moreover, work-related stressors may continue to affect health even after exposure has ended. Empirical evidence suggests, for example, that exposure to unfavourable working conditions during midlife may correlate with health during retirement in later life [34], which would indidcate that there is some persistence of work-related health impairments. But the drivers of this association are not fully understood. Apart from persistent health shocks, persistence in working conditions or unobserved heterogeneity that influences positioning in the job quality spectrum, as well as health outcomes, are further conceivable mechanisms.

Accounting for unobserved heterogeneity and reverse causality has been shown to generally reduce the association between work and health [1], whilst addressing endogenous selection into economic inactivity tends to increase the estimated health impacts of work [29]. Disentangling the contribution of individual characteristics from work-related effects among older workers will help to gauge the potential health benefit that better working conditions may deliver during the final career stages.

By analysing the effects of job quality on multiple health outcomes in a cross-national representative panel of older workers, this paper contributes to the wider literature on work and well-being. In combining earnings, psychosocial job characteristics and job insecurity, it analyses the different pathways through which work may affect physical health, mental well-being as well as functional disabilities. Longitudinal data from SHARE allows accounting for time-invariant unobserved heterogeneity, model potential reverse causality and examine the persistence of work-related health effects over time. In conjunction with tests and correction for endogenous sample selection, this paper examines to what extent good jobs and good pay determine good health among older workers in European countries now and over time.

The remainder of the paper is organised as follows: Section two introduces the data and variables for the analysis. The empirical model is described in Section three. Section four reports the key findings, before Section five concludes.

## Data, variables and summary statistics

### Dataset

The Survey of Ageing, Health and Retirement in Europe is a large, representative, cross-nationally comparable panel-survey with data on health, socio-economic characteristics and the labour market status, including job quality instruments of people aged 50 years and older and their partners [35, 36]. SHARE was launched in 2004 and has been conducted on a biannual rhythm since. To date, the survey has collected information on more than 110,000 individuals from 20 European countries and Israel over five waves. The first wave was fielded in 2004 in 11 northern, western and southern European countries. For the second wave in 2006/2007, Poland and the Czech Republic joined the survey. The third wave, SHARELIFE, adds individual retrospective life history data. The fifth and most recent wave covers the years 2012/2013. Though it did not include job quality information, it covers health and collects key retrospective data on socio-economic circumstances and healthiness during childhood. For more information on the data collection and survey design see the technical reports [37–41].

This study uses an unbalanced panel of employed people aged 50–65 years in 15 countries from sweeps one to five with complete information on all covariates. The list of covered countries comprises Austria, Belgium, Czech Republic, Denmark, Estonia, France, Germany, Greece, Italy, the Netherlands, Poland, Slovenia, Spain, Sweden and Switzerland. Data from wave three (SHARELIFE) contains retrospective life-course data, but only a very limited selection of contemporaneous health measures and is therefore not usable for the analysis of concurrent health effects. Wave 5 did not infer job quality data, but covers health and other background covariates. The number of observations changes with model specification.^1^

### Variables

#### Health outcomes

The Survey of Ageing, Health and Retirement in Europe collects information on the prevalence of severe and mild chronic conditions, mobility limitations, functional limitations, mental well-being and self-assessed health. These pieces of information have been grouped and combined into several indicators of physical and mental health.

Acute health conditions (AHC) is defined as a binary variable that indicates whether somebody has been diagnosed with a heart attack, stroke or cancer. Cardiovascular risks (CVR) counts diagnoses of hypertension, high blood cholesterol and diabetes. The index of musculoskeletal disorders (MSD) is a sum score of mobility limitations resulting from impaired functioning of the musculoskeletal system, such as physical difficulties with walking, sitting, climbing stairs, or carrying and lifting, and is an indicator of joint and muscle pain. Mental health (MH) is measured by the EURO-D scale from 12 self-reported items. EURO-D has been validated as a cross-national scale of mental health in elderly populations [42, 43] and has been shown to correlate with clinical depression [44, 45]. Functional disabilities (FD) measures the difficulties a person has with self-care (ADL) and household management (IADL) in 13 activities. The scale is dichotomised to distinguish between people with any functional disabilities from those with none. And finally, self-assessed general health (SAH) summarises an individual’s overall health status in five categories ranging from “excellent”, to “very good”, “good”, “fair” and “poor”. Self-reported health is an ordinal variable. Acute conditions and functional disability are measured by binary variables. Indices of cardiovascular risk factors, musculoskeletal disorders and mental health are counts of the underlying binary items. Table 4 in the Appendix reports summary statistics of the derived health indices and lists included variables.

#### Job quality

Job quality is derived from instruments on psychosocial working conditions and job prospects from the job content questionnaire [46] and the effort-reward imbalance questionnaire [47] as well as supplementary data on earnings, occupation, industry and the employment contract type. Following the concept outlined in Green et al. [8] and Green and Mostafa [16], the items are grouped into three key domains: intrinsic job quality, job insecurity and earnings. Earnings capture the material dimension of job quality. Job insecurity combines risk of job loss with career prospects. Intrinsic job quality includes most of the commonly explored aspects of workplace quality grouped into skill use and discretion, social environment, physical environment and work intensity. The resulting domains resemble recent OECD efforts to measure cross-country differences in work quality [48]. Scores are calculated using principal component analysis (PCA) in the pooled cross-sectional dataset.

Intrinsic job quality combines occupational skill-levels with items on the opportunity to develop new skills, discretion over how to do the work, support in difficult situations, physical work demands and time pressure. The psychosocial measures are coded on 4-point Likert-scales ranging from 1 “Strongly Agree” to 4 “Strongly Disagree”. To improve cross-national comparability of the items, responses are dichotomised to distinguish between agreement and disagreement. A measure of intrinsic job quality based on the full range of responses is used in robustness checks. Occupations are dichotomised along skill levels into a group of graduate occupations (managers, professionals), advanced vocational occupations (technicians and associate professionals), semi-skilled occupations (clerks, service workers, skilled agricultural workers, craft workers, plant and machine operators) and elementary occupations. In doing so, occupational skill-levels are assumed to be similar across countries. Semi-skilled occupations are chosen as the reference category. Intrinsic job quality is derived from the first component of a principal component analysis over the discussed list of items. Higher values indicate better jobs.

Job insecurity is measured by a combination of information on job security and career prospects as well as type of employment contract and the estimated risk of unemployment. Perceptions of job security and career prospects are assessed as part of the battery on psychosocial work stressors. Responses are again dichotomised to measure agreement versus disagreement. Working on a fixed-term contract is assumed to increase job insecurity and thus added as an indicator. The prospective individual risk of unemployment is predicted within the longitudinal sample of people in work from a set of probit models of unemployment in $t + 1$ conditional on sex, 5-year age-groups, education, period dummies, a set of industry dummies and country effects separately for each wave. The predicted probability of unemployment is dichotomised at the 75th percentile to capture high prospective risks of joblessness. The first component from a PCA of the four items is thought to summarise job insecurity. Higher values correspond to worse job prospects and higher insecurity.

Data on annual labour earnings was converted to average monthly earning using information on the number of months worked in the job and then transformed to German prices in 2005 by purchasing power parity conversion rates. To limit the potential bias from item non-response, the data includes imputed income values. SHARE uses a multiple imputation procedure drawing on longitudinal information, a set of concurrent variables and unfolding brackets to predict a range of plausible annual monetary values. The analysis uses the first plausible value.

The intrinsic job quality and job insecurity scales are *z*-standardised for ease of interpretation. Table 5 in the Appendix summarises the variables that were used in the construction of the job quality domains, the derived PCA loadings and the bivariate correlation of the facets with job satisfaction. Greater intrinsic quality and earnings correlate significantly with better job satisfaction, whilst higher job insecurity is associated with lower job satisfaction.

#### Other covariates

The full list of covariates on individual background are summarised in Table 6 in the Appendix. Besides a common list of socio-demographic covariates such as age, sex, educational attainment, marital status and country of birth, the analysis leverages data on cognitive abilities, past smoking behaviour, home ownership as well as information on socio-economic circumstances and healthiness during childhood from SHARELIFE.

Data on memory functioning, verbal fluency and numeracy is collected in short standardized tests during the main interview. Memory functioning is assessed through an immediate and delayed recall exercise of 10 words. The memory score counts the overall number of correctly recalled words ranging from 0 to 20. Fluency is given by the number of animals a respondent can name within a minute. Numeracy ranges from 1 to 5 and measures the number of correctly solved mathematical exercises from a small set of basic to more involved problems. The analysis will make use of information at survey baseline, since data on numeracy and fluency were collected only during the first interview. Cognitive abilities may correlate with the individual’s positioning in the job quality spectrum.

A dummy of home ownership distinguishes between respondents who own their home outright and those that either pay off mortgages or live in rented accommodation. The indicator is thought to approximate differences in household wealth and thus financial resources to fully or partially retire from work.

An indicator of past smoking approximates health-relevant behaviour. It is one if respondents have ever smoked daily for a period of at least a year. Smoking is the leading cause for a range of morbidities and may approximate health behaviour and attitude towards risk more generally.

Information from SHARELIFE on health and socio-economic circumstances during childhood allow for examining the effect of usually unobserved determinants of job selection and health risks. Initial healthiness is modelled by self-assessed health, a dummy for whether respondents missed more than a month of school because of ill-health, and an average over a range of acute and chronic health conditions, including respiratory problems, asthma, chronic ear problems, difficulties seeing with eyeglasses, migraines, or psychiatric problems. Measures on socio-economic positioning and cognitive skills include the number of rooms per household member at age 10, the number of books at home and an indicator of self-reported skills in maths and languages at age 10. Previous research has generally confirmed the validity of these retrospective measures [49, 50]. This set of information provides the opportunity to enrich the determinants of job choice and healthiness at survey baseline.

Finally, information on early retirement ages from the European Mutual Information System on Social Protection is used to distinguish between those that are potentially eligible for early retirement pensions and those below retirement age. In countries without early retirement pathways, the normal retirement age is applied instead. Retirement decisions are often made jointly within households [51]. The proportion of retired adults in the household may thus change the individual propensity to retire. The variable is derived within the sample. I assume that neither early retirement eligibility nor the proportion of retired household members affects individual health directly but shifts the propensity to retire. Consequently, these variables can be excluded from the main model and used as instruments in a secondary equation to examine the impact of selection on the job quality-health nexus.

## Empirical model

Let $h_{it}^{*j}$ be latent health in dimension $j$ of individual $i$ at time $t$. Concurrent health in dimension $j$ is modelled to depend on time-variant and time-invariant individual background characteristics $X_{it}$, job quality ${\text{JQ}}_{it}$ and a random component capturing unobserved individual frailty $\vartheta_{i}^{j}$ and idiosyncratic health shocks $\varepsilon_{it}^{j}$

1 $$h_{it}^{*j} = \beta^{j} {\text{JQ}}_{it} + \gamma^{j} X_{it} + \vartheta_{i}^{j} + \varepsilon_{it}^{j} .$$

The error term is split into a random normally distributed time constant individual effect to measure unobserved frailty differences between individuals ($\vartheta_{i}^{j}$), and an idiosyncratic error $\left( {\varepsilon_{it} } \right)$. Random effects will pick up unobserved, time-constant differences in the risk of developing certain health conditions. Both error components are assumed to neither correlate with the independent variables nor with each other.

This baseline specification may be insufficient to identify $\beta^{j}$ if job quality is correlated with the time-constant frailty term or the idiosyncratic error due to unobserved heterogeneity, sample selection or reverse causality.

Firstly, to enhance the specification of unobserved heterogeneity, within-individual means of job quality variables are added as covariates. The frailty component changes to $\vartheta_{i}^{j} = \psi^{j} + \pi^{j} \overline{\text{JQ}}_{i} + \eta_{i}^{j}$, where $\eta_{i}^{j}$ represents random effects that are independent of the observed covariates. Including within-individual means of job quality items allows for non-zero correlation between job facets and time-constant unobserved individual factors. In other words, this specification allows for characteristics that influence positioning in the job quality spectrum as well as health. In the case of linear panel models correlated random effects are equivalent to a fixed-effects approach. For non-linear models the approach provides a more flexible alternative to conditional maximum likelihood estimation at the expense of additional parametric assumption about the distribution of unobserved heterogeneity [52]. A test of joint significance of the added within-individual means helps to decide between random effects and correlated random effects models [53]. To allow for systematic differences between the sub-panels the empirical implementation will include intercepts for each sub-panel besides a full set of period effects. Doing so accounts for potentially unobserved sample selection mechanisms that may correlate with the covariates. Retrospective information on healthiness and socio-economic positioning during childhood are added in the sensitivity analysis to test whether these background measures suffice to make random effects specifications consistent.

Secondly, endogenous sample selection is modelled through a two-step Heckman style selection approach [54, 55]. In the first step, the propensity to work is estimated conditional on a set of instruments $Z_{it}$, i.e. early retirement eligibility (age ≥ early retirement age) and the proportion of retired household members, and the other model covariates. Observing an individual in work is conditional on the propensity to work $w_{it}^{*}$ which is captured by the indicator $w_{it}$. The indicator will equal one if respondent $i$ is in work at time $t$. Selection may for instance pick up a healthy worker effect, where healthier and more resilient workers are more likely to work beyond the early retirement threshold. The selection model includes within-individual means of the instrument variables $\overline{{Z_{i} }}$ and individual constant effects $\tau_{\text{i}}$ to account for time-invariant unobserved heterogeneity that influences the timing of retirement

2 $$w_{it}^{*} = \alpha_{t} Z_{it} + \delta_{t} X_{it} + \omega_{t} \;\overline{{Z{}_{i}}} + \;\tau_{\text{i}} + u_{it} .$$

Endogenous sample selection implies that selection processes correlate with the idiosyncratic error components in Eq. (1). This can be easily tested within the extended approach.

Equation (2) is estimated by a set of probit regressions separately for each period in the pooled sample. The estimates are used to derive the inverse Mills ratio $m_{it} \left( \cdot \right)$. In the second step, the inverse Mills ratio is added to the list of covariates in Eq. (1). The inverse Mills ratios represent the nonselection hazard and will capture associations between the decision to cease work and the work-health nexus over and above the time-invariant unobservables that are already included in the main models. Significance tests can be used to decide whether sample selection biases the unadjusted estimates.

The specific choice of estimator for Eq. (1) will change with health outcomes and follows common practice in the literature. Acute health conditions (AHC), functional disability (FD) and self-assessed health (SAH) which are either dummies or ordinal variables will be estimated using logit or ordered logit models. Cardiovascular risk factors (CVR), musculoskeletal disorders (MSD) and mental health (MH) are counts of reported symptoms and will be estimated using Poisson regressions. All models include period dummies, a set of dummies for each sub-panel, and are estimated with robust standard errors that allow for heteroscedasticity and serial correlation. In the following, job quality effects on concurrent health are discussed first, before examining the persistence of job quality related health effects over time.

## Results

### Job quality effects on health levels

Table 1 reports the average marginal effects of job quality on concurrent health outcomes in the unbalanced panel of 50- to 65-year-olds across different model specifications. Specification 1 assumes conditional independence between the time-constant random effects and job quality, specification 2 relaxes this assumption, and specification 3 accounts in addition for endogenous selection into retirement. Particularly the introduction of correlated random effects changes the estimated job quality effects substantially.

**Table 1:** Average marginal effects (AME) of job quality facets on concurrent health outcomes

	(1) AHC	(2) CVR	(3) MSD	(4) MH	(5) FD	(6) SAH
Specification 1: random effects						
Intrinsic quality	–0.00018 (0.000352)	0.00168 (0.00469)	–0.0900^***^ (0.00789)	–0.142^***^ (0.0123)	–0.0048^***^ (0.000970)	–0.303^***^ (0.0219)
Insecurity	0.00032 (0.000318)	0.00968^**^ (0.00424)	0.0416^***^ (0.00698)	0.0865^***^ (0.0109)	0.00283^***^ (0.000737)	0.110^***^ (0.0194)
Monthly pay	–0.00084^**^ (0.000349)	0.0000400 (0.00426)	–0.0405^***^ (0.00737)	–0.0405^***^ (0.0112)	–0.0022^***^ (0.000732)	–0.132^***^ (0.0200)
*N*	23,116	23,116	23,128	23,110	23,125	23,128
Wald (job quality)	0.0684	0.156	0.000	0.000	0.000	0.000
χ^2^ (individual effects)	0.000	0.000	0.000	0.000	0.000	0.000
Specification 2: correlated random effects						
Intrinsic quality	–0.00054 (0.000906)	–0.00021 (0.0108)	–0.0398^**^ (0.0193)	–0.119^***^ (0.0319)	–0.000522 (0.00228)	–0.0868 (0.0543)
Insecurity	0.00138^*^ (0.000838)	–0.0042 (0.00832)	0.0192 (0.0164)	0.0687^***^ (0.0257)	0.00235 (0.00195)	0.0707 (0.0469)
Monthly pay	–0.00083 (0.000781)	0.0171^*^ (0.00925)	–0.0275^*^ (0.0165)	–0.0802^***^ (0.0253)	0.000658 (0.00178)	–0.0672 (0.0450)
*N*	23,116	23,116	23,128	23,110	23,125	23,128
Wald (job quality)	0.278	0.288	0.0369	0.000	0.656	0.0712
Hausman	0.526	0.0814	0.0100	0.215	0.0958	0.0001
Specification 3: correlated random effects + selection						
*N*	23,116	23,116	23,128	23,110	23,125	23,128
Wald (job quality)	0.381	0.259	0.0248	0.0000	0.743	0.032
Wald (Mills ratio)	0.062	0.199	0.0031	0.00034	0.538	0.569

Average marginal effects of job quality facets on health in an unbalanced panel of workers aged 50–65 in 15 European countries. Panel data estimator depending on health dimension: logit (AHC, FD), Poisson (CVR, MSD, MH), ordered logit (SAH). The interpretation of AME changes with estimator: effect on the probability (logit), effect on the number (Poisson), or effects on the linear index (ordered logit). Specification 1 is estimated by random effects, specification 2 introduces correlated random effects and specification 3 corrects for potential selection bias. Rows at the bottom of each panel report *p*-values from joint significance tests. Robust standard errors in parentheses

*AHC* (heart attack, stroke or cancer), *CVR* cardiovascular risk factors, *MSD* musculoskeletal disorders, *MH* mental health, *FD* functional disabilities (ADL and IADL), *SAH* self-assessed health

* *p* < 0.1, ** *p* < 0.05, *** *p* < 0.01

In general, there are systematic inter-individual differences in health across job quality, individual background characteristics and the unobserved frailty component. Though not shown in the table, smoking habits correlate with worse health across all examined dimensions. There is also evidence for an educational gradient in physical health. Cognitive abilities correlate significantly with better health across some dimensions. Furthermore, there are significant differences in health by sex and age and there is substantial variation in the prevalence of health conditions across countries. Rarer conditions such as acute health conditions and functional disabilities are generally less clearly associated with observed individual characteristics.

Results from specification 1 confirm inequities in job quality correlate with inequities in health. Older workers in better jobs (better intrinsic quality, lower insecurity, and higher pay) report significantly lower levels of MSD, better MH, a lower probability of FD and better SAH. The directions of the correlations follow theory and intuition: higher intrinsic job quality, lower job insecurity and greater pay correlate with fewer health complaints. Only the prevalence of CVR is not statistically significantly associated with joint job quality, though there is a significant individual effect of job insecurity. Generally, job quality facets correlate with health outcomes in tandem: either none or all are statistically significant. But there are exceptions such as an isolated effect of pay on acute conditions and the discussed effect of job insecurity on CVR.

Though statistically significant, the differences in a specific health dimensions along the job quality spectrum are quantitatively moderate. Relative to the mean, an increase in intrinsic job quality by a standard deviation would for example lower the number of MSD by roughly 9% *(β* = –0.090/mean = 1.034) and improve the mental health score by around 7% (*β* = –0.142/mean = 1.924). As argued above, the association between job quality facets and health may be partially spurious. Unobserved individual characteristics could determine job quality as well as health. Specification 2 thus introduces correlated random effects to account for this potential relationship of unobserved individual frailty with job characteristics. A joint test for significance of the added within-individual means reveals whether specification 1 has to be rejected.

The robust Hausman tests reject random effects for CVR, MSD, FD and SAH at the 10% level of significance or lower (specification 2). For AHC and MH, there is no clear indication for a violation of the random effects assumption. Especially job quality effects on mental well-being are hardly affected by the introduction of unobserved heterogeneity.

Allowing for correlated random effects tends to generally reduce job quality effects on health. The drop in relative effect size is overall largest for intrinsic job quality and smallest for pay. For example, the average marginal effect of intrinsic job quality on the number of MSD moves from –0.09 to –0.04 between specifications. In other words, if we were to improve intrinsic job quality for 250 workers by a standard deviation we would expect around ten fewer reported musculoskeletal symptoms. But, for a small number of cases, estimates increase after the introduction of correlated random effects. An increase in job insecurity is, for example, found to affect the probability of acute health conditions. Earnings growth seems more strongly associated with mental health than levels of earnings. And although only weakly statistically significant, higher pay may increase the chance of developing cardiovascular risk factors.

Not reported separately, but conditional fixed-effects Poisson models instead of the correlated random-effects specification do not alter the conclusions for the count outcomes (CVR, MSD, MH). Neither does the addition of retrospective childhood background variables change the findings or the significance of the correlated random effects, nor can job quality measures, based on the full range of responses, provide a richer picture.

However, if individuals who suffered work-related health shocks selected out of the sample over time, specification 2 could underestimate the consequence of low job quality on health. Specification 3 adjusts and tests for endogenous sample selection. In the first stage, I run separate probit models to estimate the individual propensity to be in work for each wave. The instruments, early retirement eligibility and the fraction of retired household members, are strongly associated with the propensity to maintain work. From the estimates I predict the inverse of the Mills ratio. In the second step, specification 2 is re-estimated with the inclusion of the predicted inverse Mills ratio. The estimates confirm endogenous selection bias for a subset of health outcomes (AHC, MSD and MH). For these outcomes, cessation of work or the hazard of nonselection is associated with lower health. Nonetheless, estimated job quality effects are virtually unchanged compared to specification 2 and therefore not separately reported.

In all, better jobs with higher pay, greater intrinsic job quality and lower insecurity can help to improve mental well-being, prevent the development of musculoskeletal disorders and thus protect general health. Musculoskeletal disorders, depression and anxiety are the most frequently reported work-related health impairments in Europe [56] and based on the selection model associated with labour force participation. The positive effect of earnings on cardiovascular risk factors also highlights that the relation between job quality features and health outcomes can be complex and not strictly mono-directional. But since the effect is only significant at the 10%-level more research may be needed to establish its direction with greater confidence. Though a substantial fraction of the health differences associated with intrinsic job quality are explained by unobserved heterogeneity, the effects on mental and physical well-being and consequently general health could be genuine. Job insecurity has a clear albeit small effect on mental health, but the health risks from job insecurity could even be more far reaching if the effect on potentially fatal acute conditions holds (*p* < 0.1).

The overall magnitude of job quality effects on health also depends on the persistence of health deteriorations. In situations where recovery takes time or continued exposure to demanding working conditions inhibits recovery, job quality effects on health should be stronger. But a large fraction of the cross-sectional association between job quality and health seems to stem from individual risk factors that determine both the positioning in the job quality spectrum as well as health. What if individual factors also determine the dynamics of health; job quality may affect concurrent well-being without altering the underlying individual dynamics. Furthermore, correlated random effects treat pre-existing work-related conditions as an individual characteristic and not as a result of work. Static models may therefore not be able to fully capture consequences of job quality on well-being and health. The next section illuminates the effect of job quality on the dynamics of health. If the influence of job quality on health accumulates through a multiplier effect, even relatively small immediate gains may pay off over the longer run.

### The persistence of job quality effects

Persistence of work-related health effects may be evident in two ways. Firstly, work-related impairments can transmission over time through persistent health. Health can be seen as a stock that accumulates or depreciates. In this context, individuals may not fully recover from an ailment within short periods of time. Disorders such as hypertension or diabetes are, for example, chronic by nature. Health shocks could therefore develop lasting effects through a transmission from current health to future health. Secondly, if work-related health effects last, past levels of job quality should have a direct effect on current health. In other words, cumulative exposure to poor job quality should come with worse health consequences than a one-off period of work stress. In the following, I explore persistence of job quality related health effects from both angles.

To examine the contribution of job quality on health dynamics, once-lagged values of the health outcome under investigation are added to the list of covariates in Eq. (1). In combination with once-lagged job quality and lagged time-varying variables, this dynamic specification does not only reveal job quality effects over and above the individual trends in health, but also addresses concerns of reverse causality from health on job quality facets that may confound contemporaneous associations. Models with lagged dependent variables introduce a possible relation between the individual frailty component $\vartheta_{i}^{j}$ and lagged health $h_{it - 1}$: the time-constant frailty component influences health and job quality before the survey had commenced. I follow common practice in the literature and parameterise this association by including initial values of health dimension $j$ and of the exogenous covariates to the frailty component: $\vartheta_{i}^{j} = \psi^{j} + \mu^{j} h_{i1}^{j} + \tau^{j} {\text{JQ}}_{i1} + \eta_{i}^{j}$ [e.g. 33]. Similar models have been used in the extant literature on working conditions and health [57, 58]. In this context, health shocks may last beyond the short term: work-related ailments reduce current health and because current health determines future health, work stressors may develop a lasting impact on health. Over time, through cumulative exposure, work-related health effects could contribute to the significant cross-sectional health inequities by job quality that are observed in the data. The evidence on persistence is indirect and assumes that the model parameters are constant as health changes.

With standard errors that allow for serial correlation, the models can be consistently estimated within the pooled dataset. Note that in this specification, individuals may have retired between $t - 1$ and $t$. In the sensitivity analysis, I test whether the addition of childhood variables to better control for initial conditions changes the conclusions.

From the estimates, there is no clear statistical indication that job quality may contribute to health trajectories beyond a single point in time (Table 2). For mental health, the coefficient of once-lagged intrinsic job quality is statistically significant at the 10%-level with the expected signs, but the estimated effects may be too small to generate persistence. From the estimates, levels of mental health are more time-variable than physical health. Adding childhood characteristics to enrich the dynamics of health and to further control for initial conditions does not change the estimates. In all, the overall evidence for persistence remains weak and inconclusive.

**Table 2:** Average marginal effects (AME) of past job quality on concurrent health

	(1) AHC	(2) CVR	(3) MSD	(4) MH	(5) FD	(6) SAH
Intrinsic quality (*t*–1)	0.00387 (0.00545)	0.0216 (0.0142)	0.0226 (0.0351)	–0.0701^*^ (0.0422)	0.00693 (0.00597)	–0.0552 (0.0462)
Insecurity (*t*–1)	–0.000108 (0.00413)	–0.0107 (0.0114)	0.0216 (0.0277)	0.0422 (0.0330)	0.00711 (0.00441)	–0.0551 (0.0385)
Monthly pay (*t*–1)	0.00292 (0.00545)	0.000397 (0.0121)	–0.0178 (0.0355)	–0.0428 (0.0384)	–0.00439 (0.00542)	0.00469 (0.0418)
*N*	14,339	14,310	14,345	14,278	14,338	14,347
Wald (job quality)	0.845	0.371	0.738	0.112	0.232	0.373

Estimates of lagged job quality facets on health in an unbalanced panel of workers aged 50–65 in 15 European countries conditional on lagged health status and background characteristics. Initial values of the time varying exogenous covariates (including job quality) and health are added as covariates to control for initial conditions. Pooled data estimator depending on health dimension: logit (AHC, FD), Poisson (CVR, MSD, MH), ordered logit (SAH). The interpretation of AME changes with estimator: effect on the probability (logit), effect on the number (Poisson), or effects on the linear index (ordered logit). Robust standard errors in parentheses

*AHC* (incidence of heart attack, stroke or cancer), *CVR* cardiovascular risk factors, *MSD* musculoskeletal disorders, *MH* mental health, *FD* functional disabilities (ADL and IADL), *SAH* self-assessed health

* *p* < 0.1, ** *p* < 0.05, *** *p* < 0.01

The second approach instead enriches the dynamic structure of job quality by adding lagged values alongside current values of job quality to Eq. (1). Whereas before job quality effects were assumed to work through persistent health, here I assess whether past job quality has, conditional on the current values, a spill-over effect on current health outcomes. If job quality effects last beyond the current period, past job quality should affect current health directly. To test this hypothesis, I estimate pooled models of Eq. (1) which include the concurrent and lagged job quality values. Table 3 summarises the estimated average marginal effects.

**Table 3:** Dynamics of job quality and health. Average marginal effects

	(1) AHC	(2) CVR	(3) MSD	(4) MH	(5) FD	(6) SAH
Intrinsic quality (*t*)	0.000316 (0.00559)	–0.00761 (0.0148)	–0.107^***^ (0.0279)	–0.181^***^ (0.0370)	–0.00558 (0.00477)	–0.185^***^ (0.0421)
# (*t*–1)	0.00117 (0.00587)	–0.00487 (0.0156)	0.00748 (0.0296)	–0.0151 (0.0390)	–0.00160 (0.00524)	–0.0226 (0.0437)
Insecurity (*t*)	0.000723 (0.00492)	0.00745 (0.0133)	0.0408 (0.0260)	0.0876^***^ (0.0328)	0.00676^*^ (0.00404)	0.0266 (0.0374)
# (*t*–1)	–0.00309 (0.00448)	–0.0149 (0.0133)	0.00380 (0.0246)	–0.0521 (0.0321)	0.00232 (0.00367)	–0.0420 (0.0368)
Monthly pay (*t*)	–0.000469 (0.00521)	0.0281^**^ (0.0140)	–0.0377 (0.0262)	0.0459 (0.0370)	0.00184 (0.00439)	–0.0437 (0.0399)
# (*t*–1)	0.00550 (0.00513)	–0.00498 (0.0140)	0.0129 (0.0260)	0.00709 (0.0361)	–0.000340 (0.00413)	–0.0301 (0.0400)
*N*	4247	4247	4248	4243	4248	4248
*F *(job quality, *t*)	0.999	0.207	0.000	0.000	0.231	0.000
*F* (job quality, *t*–1)	0.627	0.691	0.949	0.423	0.913	0.565

Estimates of current and lagged job quality facets on health in an unbalanced panel of workers aged 50–65 in 15 European countries conditional on background characteristics. Pooled data estimator depending on health dimension: logit (AHC, FD), Poisson (CVR, MSD, MH), ordered logit (SAH); effect on the probability (logit), effect on the number (Poisson), or effects on the linear index (ordered logit). Robust standard errors in parentheses

*AHC* (incidence of heart attack, stroke or cancer), *CVR* cardiovascular risk factors, *MSD* musculoskeletal disorders, *MH* mental health, *FD* functional disabilities (ADL & IADL), *SAH* self-assessed health

* *p* < 0.1, ** *p* < 0.05, *** p < 0.01

Firstly, as above, older workers in better jobs tend to be in better health. Secondly, past levels of job quality are not associated with current health. Past levels of job quality may determine current working conditions and current working conditions may affect health, but none of the estimates suggest that these health effects persist and accumulate over time. Workers who were exposed to low intrinsic job quality in two consecutive periods did not face greater health risks than workers whose intrinsic job quality dropped between *t* – 1 and *t*. As in Table 2, there is no clear evidence that job quality effects persist beyond a single period.

The total health gains from better jobs depend on the persistence of stressful work and work-related health impairments. The estimates suggest that among older workers in Europe, job quality related health deteriorations do not tend to develop into chronic impairments, but rather lead to temporarily worse mental well-being, increased musculoskeletal complaints and thus transitional spells of poor general health. Exposure to differential job quality does not contribute to growing health inequities across the job quality distribution among older workers. Job stress for the average worker may be temporary with sufficient recovery time to recuperate. Existing mechanisms such as sick-leave, workplace health promotion programmes, or job change may be sufficient to prevent transitional spells of ill-health developing into chronic impairments for the average worker aged 50+ in the selected countries. Removing work stress through better jobs may therefore contribute to direct improvements of certain health dimensions, but with little additional gains in the medium or long term.

## Discussion and conclusions

This study examines the effects of job quality on a range of physical and mental health outcomes in an unbalanced panel of workers aged 50–65 years across 15 European countries. By applying a multi-facetted concept of job quality, the analyses shed light on different pathways through which work can affect physical and mental health in the short term and beyond. Unobserved heterogeneity, endogenous sample selection and reverse causality may bias the cross-sectional relation of job quality with health and well-being. Addressing these issues, I examine to what extent good pay and good jobs lead to good health.

Three themes emerge from the estimates. Firstly, inequities in job quality correlate with inequities in health. Musculoskeletal disorders, mental well-being, functional disabilities and general health worsen as we move down the job quality ladder. There is even evidence to suggest that joint job quality and especially pay are associated with the occurrence of potentially fatal acute conditions. Except for a few cases, job quality facets correlate with health outcomes in tandem. The health gradient by job quality is quantitatively moderate on the individual level, but may sum up to a substantial number of avoidable conditions at population level. Health differences by level of income and job insecurity further widen work-related health inequities. Modelling job quality by a single facet would have underestimated these differences.

Secondly, a substantial proportion, but not all, of the cross-sectional correlation between job quality and health outcomes is accounted for by unobserved heterogeneity. Especially health inequities by intrinsic job quality may reflect more inter-individual differences than work-related consequences, whereas pay and job insecurity effects change less when allowing for correlated random effects. Though the effect sizes are overall modest, better jobs can help to improve individual mental well-being and reduce musculoskeletal complaints—two key work-related health dimensions. Despite evidence for a healthy worker effect on sample composition, i.e. economically inactive individuals tended to be in worse health, there was no indication that this affected the job quality-health nexus.

Thirdly, job quality related health changes appear to be transitional for the average older worker. There is no clear indication that effects last and accumulate beyond a single period. Workers who were in low quality jobs in consecutive data sweeps were not in worse health than workers who transitioned into a low quality job between sweeps. Thus, differences in job quality do not seem to contribute to a further widening of work-related health inequities among older workers in Europe. Existing mechanisms of social protection, health care and prevention may be sufficient to support full recovery from work-related ailments for the average worker within relative short periods of time. Findings from the selection model confirm that differences in the propensity to retire do not affect the relationship of job quality with health. This dovetails with the transitional nature of work-related health impairments. If workers can recover from ailments and disorders within employment, retirement is a less essential mechanism to recuperate health.

However, some caveats have to be borne in mind when interpreting the findings. The job quality model by Green et al. conceptualises working time quality as an additional facet of job quality, but for which SHARE does not provide information. The ability to flexibly change, for example, the number of working hours or the work schedule may be of special importance for older workers who have to accommodate health impairments or family care responsibilities. Job-quality related effects on health and well-being may thus not be captured fully. It is also conceivable that by focussing on job quality and not specific work stressors, the approach is conceptually too far removed from well-being and introduces confounding influences. However, the measured dimensions of job quality in the analysis share largely the same set of information that would have been used to measure psychosocial work stressors (control, effort and rewards). Furthermore, the panel is relatively short and unevenly spaced since wave 3 includes only limited contemporary information. More data would have allowed estimation of determinants of rarer conditions with greater confidence and would have enriched the study of health dynamics. Though selection into retirement did not emerge as a critical confounder, there are other sources of endogeneity that could still bias the estimated relations. For example, workers may have sufficient discretion to mould their job to their needs after, for example, a health shock. The flexibility to recover in work from health shock would be a key dimension of job quality among older workers and warrants further investigation.

In all, the results support the importance of recent efforts by the OECD, the European Foundation for the Improvement of Working Conditions and the European Commission to bring intrinsic job quality to the forefront of the policy debate [59–61]. Bad jobs may create negative externalities in the form of greater health care expenses for mental health problems and musculoskeletal disorders, increase sickness absence and induce productivity losses. Better jobs would directly help to limit the incidence of avoidable musculoskeletal disorders and deteriorations of mental well-being, if taken to the population level. But the findings also highlight that job quality-related health impairments are transitional. Existing health inequities along the job quality ladder appear to have largely formed during earlier stages of working life. For the wider policy agenda to extend working lives, the main obstacle may rather be the sorting of people with lower health into jobs of lower quality. This range of jobs appears ill-suited to cater for further health deteriorations and thus may create additional early retirement incentives.

## Appendix

See Tables 4, 5 and 6.

**Table 4:** Health outcomes

Health indices	Variables	In work		Total	
		Mean	SD	Mean	SD
Acute health conditions (AHC)	Has a doctor ever told you that you had a heart attack including myocardial infarction or coronary thrombosis or any other heart problem including congestive heart failure (ph006d1), a stroke or cerebral vascular disease (ph006d43), or cancer or malignant tumour (ph006d10) AHC: 0/1 if any acute condition	0.078	0.268	0.121	0.326
Cardiovascular risks (CVR)	Has a doctor ever told you that you had/currently have high blood pressure or hypertension (ph006d2), high blood cholesterol (ph006d3), or diabetes or high blood sugar (ph006d5) CVR: sum score [0, 3]	0.474	0.702	0.578	0.777
Musculoskeletal disorders (MSD)	Because of a health problem, do you have difficulty walking 100 m, sitting for about 2 h, getting up from a chair after sitting for long periods, climbing several flights of stairs without resting, climbing one flight of stairs without resting stooping, kneeling, or crouching, reaching or extending your arms above shoulder level, pulling or pushing large objects, lifting and carrying weights over 5 kg, picking up a small coin from a table (ph048d1–ph048d10), pain in your back, knees, hips or any other joint (ph010d1) MSD: sum score [0, 11]	1.034	1.509	1.456	2.019
Mental health (MH)	Feels depressed, no hopes for the future, suicidal, feels guilty, trouble sleeping, less interest in things, irritable, diminished appetite, fatigue, difficulty concentration on entertainment or reading, lack of enjoyment, tearfulness (euro1–euro2) MH: sum score [0, 12]	1.924	1.915	2.204	2.145
Functional disability (FD)	Difficulty with dressing, walking across a room, bathing or showering, eating, getting in or out of bed, using the toilet, using a map, preparing a hot meal, shopping, making telephone calls, taking medications, doing work around the house or garden, managing money (ph049d1–ph04913) FD: 0/1 if any limitation	0.065	0.246	0.117	0.322
Self-assessed health (SAH)	Would you say your health is 1. Excellent, 2. Very good, 3. Good, 4. Fair, or 5. Poor (sphus)	2.665	0.998	2.906	1.061

**Table 5:** Job quality facets and their association with job satisfaction

Dimension	Variables	PCA loading	Correlation with job satisfaction
Intrinsic quality	job is physically demanding (disagree = 1/agree = 0)	0.4210	0.3108^***^
	constant time pressure due to a heavy workload (disagree = 1/agree = 0)	0.1186	
	very little freedom to decide how I do my work (disagree = 1/agree = 0)	0.4117	
	opportunity to develop new skills (agree = 1/disagree = 0)	0.4833	
	adequate support in difficult situations (agree = 1/disagree = 0)	0.2730	
	Occupational skill level: higher tertiary (1/0)	0.3942	
	Occupational skill level: post-secondary (1/0)	0.0849	
	Occupational skill level: primary (1/0)	–0.4104	
Job insecurity	Poor prospects for job advancement (agree = 1/disagree = 0)	0.4414	–0.2052^***^
	Poor job security (agree = 1/disagree = 0)	0.6450	
	Fixed-term contract (1/0)	0.5301	
	High unemployment risk	0.3079	
Earnings	Annual gross income from employment previous year (ydipv), annual gross income from self-employment previous (yindv), ep014, exrate		0.0743^***^

The unemployment risk was estimated using a probit model of the transition from work to unemployment between survey waves conditional on age, age squared, gender, educational attainment, industry, survey period, and a set of country dummies. The predicted probability was dichotomised at the 75% percentile to indicate high prospective unemployment risk. Number of observations: 41,173 (intrinsic quality), 25,763 (job security). Correlation with job satisfaction (satisfied with job: 1 “strongly disagree”, 2 “disagree”, 3 “agree”, 4 “strongly agree”)

*** *p* < 0.01

**Table 6:** Individual background

Variables	Mean	SD	Min	Max
Individual background (*N* = 23,128)				
Age 50–54	0.454	0.498	0	1
Age 55–59	0.375	0.484	0	1
Age 60–65	0.170	0.376	0	1
Female	0.489	0.500	0	1
Foreign born	0.087	0.282	0	1
Home owner	0.425	0.494	0	1
Married/in partnership	0.768	0.422	0	1
Education: lower secondary or less	0.259	0.438	0	1
Education: upper secondary	0.419	0.493	0	1
Education: tertiary	0.321	0.467	0	1
Numeracy (at baseline)	3.790	0.983	1	5
Memory (at baseline)	10.304	3.192	0	20
Verbal (at baseline)	22.741	7.376	0	100
Ever smoked	0.555	0.497	0	1
Childhood background (*N* = 17,100**)**				
Poor/fair health	0.080	0.271	0	1
Good health	0.231	0.421	0	1
Very good/excellent health	0.689	0.463	0	1
Health conditions	0.018	0.038	0	0.4
Missed school	0.123	0.329	0	1
Rooms per hh member (age 10)	0.809	0.420	0	11.25
1 + Bookshelf (age10)	0.755	0.430	0	1
Relative skills (age 10)	0.003	0.054	0	1
Not in school (age 10)	0.382	0.732	–2	2

The covariates include additional sets of country and period dummies as well as dummies for sub-panels by initial interview wave and dropout sweep

## Footnotes

1 The total SHARE sample includes almost 207,000 person–year observations. Removing wave 3 deletes 27,908 observations; restricting the sample to the age-bracket 50–65 shrinks the sample by a further 87,738 observations. Many participants did not respond at follow-up interviews and provide only one interview. Questionnaire routing for some items reduce the number of observations further. Respondents in partial retirement were not asked about their career prospects or job security. In practice the number of observations varies between around 23,100 for concurrent health effects and 13,800 for health dynamics.
